# Towards combinatorial targeted therapy in melanoma: From pre-clinical evidence to clinical application (Review)

**DOI:** 10.3892/ijo.2014.2491

**Published:** 2014-06-10

**Authors:** GIULIA GRAZIA, ILARIA PENNA, VALENTINA PEROTTI, ANDREA ANICHINI, ELENA TASSI

**Affiliations:** Human Tumors Immunobiology Unit, Department of Experimental Oncology and Molecular Medicine, Fondazione IRCCS Istituto Nazionale dei Tumori, 20133 Milan, Italy

**Keywords:** melanoma, combinatorial treatment, targeted therapy, apoptosis

## Abstract

Over the last few years, clinical trials with BRAF and mitogen-activated protein/extracellular signal-regulated kinase (MEK) inhibitors have shown significant clinical activity in melanoma, but only a fraction of patients respond to these therapies, and development of resistance is frequent. This has prompted a large set of preclinical studies looking at several new combinatorial approaches of pathway- or target-specific inhibitors. At least five main drug association strategies have been verified *in vitro* and in preclinical models. The most promising include: i) vertical targeting of either MEK or phosphoinositide-3 kinase (PI3K)/mammalian target of rapamycin (mTOR) pathways, or their combined blockade; ii) association of receptor tyrosine kinases (RTKs) inhibitors with other pro-apoptotic strategies; iii) engagement of death receptors in combination with MEK-, mTOR/PI3K-, histone deacetylase (HDAC)-inhibitors, or with anti-apoptotic molecules modulators; iv) strategies aimed at blocking anti-apoptotic proteins belonging to B-cell lymphoma (Bcl-2) or inhibitors of apoptosis (IAP) families associated with MEK/BRAF/p38 inhibition; v) co-inhibition of other molecules important for survival [proteasome, HDAC and Signal transducers and activators of transcription (Stat)3] and the major pathways activated in melanoma; vi) simultaneous targeting of multiple anti-apoptotic molecules. Here we review the anti-melanoma efficacy and mechanism of action of the above-mentioned combinatorial strategies, together with the potential clinical application of the most promising studies that may eventually lead to therapeutic benefit.

## 1. Introduction

Metastatic melanoma is the leading cause of death from skin cancer, with a 5-year survival rate of less than 10%, and its incidence has been continuously increasing in the last decades (1). Before 2011 the FDA approved treatments for metastatic melanoma were dacarbazine, recombinant human interleukin (IL)-2 and high dose or pegylated interferon (IFN)-α. Collectively, these treatments yielded tumor responses only in around 20% of patients, often with no benefit on progression-free survival (PFS) or overall survival (OS) (2).

In 2002 Davies *et al* identified BRAF somatic missense mutations in 66% of malignant melanomas; in 80% of cases it was a single substitution (V599E) within the kinase domain, which resulted in a constitutively active protein (3). This discovery rapidly led to the development of a selective mutant-BRAF-inhibitor, vemurafenib (PLX4032), which in an initial phase I study led to a response rate of 81% in melanoma patients, and in a randomized phase III clinical trial showed a significant increased efficacy compared to dacarbazine treatment: OS at 6 months was 84% in the vemurafenib group and 64% in the dacarbazine group, while the PFS were 5.3 and 1.6 months, respectively (4,5). As a consequence of these results, vemurafenib was the first oral BRAF inhibitor approved by the food and drug administration (FDA) in 2011 for the treatment of melanoma. A different BRAF inhibitor, dabrafenib (GSK2118436), and the MEK1/2 inhibitor trametinib (GSK1120212) were subsequently developed, and in phase III clinical studies showed improved response rates compared to chemotherapy: the median PFS was 5.1 months for dabrafenib and 2.7 months for dacarbazine (6); in trametinib trial, this compound led to a median PFS of 4.8 months and 81% 6-months OS compared with, respectively, 1.5 months and 67% in the chemotherapy (dacarbazine or paclitaxel) group (7). These results led to dabrafenib and trametinib approval by FDA for melanoma treatment between 2012 and 2013.

Although vemurafenib, dabrafenib and, to a lesser extent, trametinib were associated with impressive clinical results (in the initial trials response rates were 48–53, 50 and 22%, respectively), the majority of patients relapsed quite rapidly, as the median duration of responses was 6.7 months for vemurafenib and 5.5 months for both dabrafenib and trametinib. Furthermore, a significant percentage of patients showed intrinsic resistance (5–8). Several mechanisms of intrinsic or acquired resistance to RAF/MEK inhibitors were then elucidated: in most cases extracellular signal-regulated kinases (ERK) signaling results reactivated due to alterations that promote RAF stimulation (e.g., NRAS mutations, CRAF overexpression and RTK activation); whereas other mechanisms of resistance bypass the dependence of the tumor on RAF through, for example, MEK mutations or the overexpression of the mitogen-activated protein kinase (MAPK) agonist COT (9,10).

Besides BRAF/MEK pathway, other molecular processes are determinant for melanoma onset and progression, and might mediate intrinsic or acquired resistance to BRAF/MEK inhibitors (11). This knowledge has prompted a large set of preclinical studies looking at several new combinatorial approaches of pathway- or target-specific inhibitors.

In this review, we summarize the main survival pathways important for melanoma initiation and progression, the more relevant co-targeting strategies that have been evaluated *in vitro* or in animal models and their mechanism of action, together with the potential clinical application of the most promising studies.

## 2. Apoptosis pathways and melanoma resistance to cell death

MEK and BRAF^V600E^ inhibitors exert their anti-neoplastic effect mainly by inducing tumor cell death and modulating several molecules of the apoptotic cascade (12,13). Unfortunately, resistance to apoptosis is one important hallmark of melanoma (14), and its reversal is a common goal across most preclinical combinatorial target therapy studies, as it could lead to the overcome of primary and secondary resistance mechanisms.

In tumor cells, apoptosis is controlled by two main signaling pathways: the mitochondrial-dependent intrinsic pathway and the extrinsic cascade; their stimulation leads to the cleavage, and hence activation, of the effector caspase-3 and -7, and ultimately to apoptotic cell death.

The intrinsic apoptosis pathway is induced by cellular events, such as DNA damage, and is mediated by mitochondrial depolarization; this induces the release in the cytosol of cytochrome c, which promotes caspase-9 cleavage and the subsequent activation of effector caspases, and of the pro-apoptotic protein Second mitochondria-derived activator of caspases/direct IAP-binding protein with low pI (Smac/DIABLO). The Bcl-2-family proteins are a group of molecules, related by structure and function, which play a key role in the regulation of intrinsic apoptosis. They include: a) ‘executioner proteins’ (the pro-apoptotic members Bax and Bak), that promote the formation of mitochondrial pores, mediating the release of cytocrome c and Smac/DIABLO; b) anti-apoptotic members (Bcl-2, Bcl-X_L_, Mcl-1, Bcl2-A1 and Bcl-w), that block the oligomerization of Bax and Bak, inhibiting their activity and protecting the cell from apoptosis; and c) pro-apoptotic regulators, containing only the BH3 domain (the BH3-only proteins Bim, Bik, Bad, Bid, Bmf, Puma, Noxa and Hrk), that sense cellular stress and move to mitochondria in response to apoptotic signals, inducing the release of pro-apoptotic proteins by interacting with other members of the Bcl-2 family (15).

The extrinsic pathway of apoptosis is initiated by the engagement of death receptors [Fas/CD95, tumor necrosis factor-receptor (TNF-R)1/R2, TNF-related apoptosis-inducing ligand-receptor (TRAIL-R)1/DR4, TRAIL-R2/DR5 and TRAMP/DR3], which are members of the TNFRs superfamily and are characterized by a cytoplasmic region, known as the ‘death domain’, that enables the receptors to induce cytotoxic signals when recruited. Ligand binding results in receptor aggregation and recruitment of adaptor proteins, which, in turn, initiates a proteolytic cascade by recruiting and activating initiator caspase-8 and -10 (16).

Another group of molecules is involved in the regulation of the apoptotic process: the IAP family proteins, which inhibit caspase enzymatic activity. There are eight mammalian IAPs: neuronal apoptosis inhibitory protein (NAIP), cellular inhibitor of apoptosis (cIAP)-1, cIAP-2, X-linked inhibitor of apoptosis (XIAP), Survivin, Apollon, IAP-like protein-2 (ILP-2) and ML-IAP/Livin; many of them have been implicated in melanoma resistance to different therapies (17).

In this review, we describe pre-clinical studies that clarify how the modulation of most of the molecules involved in either intrinsic or extrinsic apoptosis pathways is critical for the efficacy of combinatorial target therapy treatments.

## 3. Parallel and vertical co-targeting of the MAPK and PI3K pathways

The RAS/RAF/MEK/ERK and PI3K/v-Akt murine thymoma viral oncogene (Akt)/mTOR pathways are the two major signal transduction cascades that are often hyper-activated in various human cancers, including melanoma (18,19). In non-neoplastic cells, these pathways can be activated following the interaction between a ligand with its receptor, such as growth factors binding to their cognate RTK (as will be described in the next section), or cytokines, including those frequently expressed in the tumor microenvironment (e.g., transforming growth factor-β and IL-6) (20,21), or even by integrins after cell-cell or cell-extracellular matrix proteins contact (22).

Alternatively, these pathways can be constitutively activated in melanoma cells due to somatic mutations of their components [e.g., BRAF^V600E^, NRAS^Q61R^, Akt1/3^E17K^, Akt1^Q79K^, or point missense mutations, insertions and deletions in the coding region of the tumor suppressor gene phosphatase and tensin homolog (PTEN)] (3,23–26), or by epigenetic modifications of their downstream effectors, like PTEN methylation (26). In recent years, it has been discovered that these genetic lesions, together with hyper-activation of RTKs, deregulated expression of RTKs ligands and aberrant expression of integrins and cytokines, contribute to tumor cell proliferation, survival and resistance to cell death (22,27–30).

The stimulation of the MAPK pathway induces RAS to interact with RAF family kinases (ARAF, BRAF and CRAF), leading to the phosphorylation and activation of MEK1/2 and ERK1/2. Subsequently, ERK interacts with multiple downstream effector proteins to promote cell cycle progression, cell survival, and other processes involved in both normal cellular physiology and transformation (31). PI3K, instead, leads to the activation of the serine-threonine kinase Akt that, in turn, recruits downstream effectors, such as mTOR, and consequently ribosomal protein S6 kinase 1 (S6K1) and eukaryotic translation initiation factor 4E binding protein 1 (4EBP1) (19,32).

MAPK and PI3K pathways not only act independently to each other, but often cross-interact, by reciprocal regulation through positive or negative feedbacks, and finally converge in the activation of common downstream proteins [such as forkhead box O (FOXO) and c-myc transcription factors, Bad and the serine-threonine protein kinase glycogen synthase kinase (GSK) 3]. For example, hyper-activated MAPK pathway can induce PI3K and its cascade by RAS, or the inhibition of the PI3K pathway can induce a rebound increase on phosphorylation of MAPK pathways molecules, such as ERK (33,34). Because of the interplay between these pathways, monotherapy directed to a single target of either signaling cascade gave only partial positive results both *in vitro* and *in vivo*, and patients enrolled in clinical trials often developed acquired resistance. This evidence gave the rationale for testing either the simultaneous targeting of several molecules of the same cascade, known as ‘vertical targeting’, or the dual blockade of both signaling pathways (35).

### Vertical co-targeting of the PI3K/mTOR pathway

Aberrant activation of the PI3K/Akt/mTOR pathway has been associated to the development of many malignant diseases, including melanoma, as a consequence of deregulation of one or more molecules involved in this pathway (36). For example, a feedback inhibition of Akt has been described following mTOR hyperactivation, possibly justifying the increased Akt phosphorylation after treatment with the mTOR inhibitor rapamycin and thus potentially hampering its efficacy (37).

For these reasons, different strategies exploiting vertical targeting with small molecules that inhibit mTOR kinase and PI3K activity are being developed and studied (Table I). A synergistic genotype- and dose-dependent anti-proliferative effect in melanoma cells was shown combining rapamycin with BKM-120, LY294002 or ZSTK474 (PI3K inhibitors), leading to cell cycle arrest and to a strong reduction of pAkt, pS6K1 and p4EBP1, but only to a modest increase of apoptosis in melanoma cell lines (38–40). A synergistic effect of the combinatorial treatment with rapamycin plus LY294002 was shown also in uveal melanoma, with the inhibition of cell proliferation and the decreased expression of cyclin D1, which has a key role in cell cycle progression (41). Werzowa *et al* (42) described the synergistic effects of the vertical inhibition of PI3K/mTOR pathway against melanoma both *in vitro* and *in vivo*. They demonstrated a marked cell cycle arrest, induction of apoptosis and inhibition of S6 and Akt phosphorylation *in vitro* combining PI-103 (a PI3K inhibitor) with rapamycin, and a statistically significant reduction in tumor volume in a human melanoma xenograft model. In addition, a synergistic effect on pAkt and pS6 was also obtained by the co-treatment with the selective PI3K p110α inhibitor GDC-0941 and rapamycin.

Taken together, these results demonstrate the critical role of the PI3K/Akt/mTOR signaling cascade in melanoma progression, further supporting the development of a combinatorial strategy to target this pathway at multiple levels.

### Vertical co-targeting of MAPK pathway

MAPK pathway is the main hyper-activated signaling cascade in melanoma, mainly as consequence of mutations in oncogenes such as BRAF and NRAS (the latter being mutated in about 20% of melanomas) (43). Under physiological conditions, the downstream effector ERK, besides promoting cell cycle progression and proliferation, controls the activation of this cascade through a negative feedback on several regulator proteins (e.g., DUSP and SPRY). In melanoma, instead, the aberrant hyper-activation of MAPK pathway leads to an enhancement of proliferative stimuli, without an efficient negative feedback caused by the atypical phosphorylation of ERK (10).

With the aim of blocking the hyper-activation of this pathway and the related uncontrolled neoplastic cell growth and dissemination, several small inhibitors have been synthesized and many strategies have been investigated to target the RAS/RAF/MEK/ERK pathway by vertical inhibition (Table II). In detail, combination of selumetinib (a MEK1/2 inhibitor also known as AZD6244) with PLX4720 (BRAF^V600E^ inhibitor) potently suppressed the colony formation of selumetinib-resistant cells and inhibited cell growth; this was associated with a synergistic decrease in pERK levels (44–46). Trametinib was combined with dabrafenib, leading to a cytostatic effect associated with diminished expression of the pro-proliferative molecules cyclin D1 and phospho-retinoblastoma (pRb) (47), and reduced skin lesions formation together with tumor growth inhibition *in vivo* (48); in addition, in dabrafenib-resistant cells, this co-treatment downregulated genes involved in cell proliferation and survival and upregulated transcripts involved in apoptosis (49). Similar results in terms of reduced *in vivo* tumor growth and improved MEK-inhibition-associated skin toxicity were obtained by co-treatment with trametinib and PLX4720 (50). An increase in apoptosis, together with the inhibition of cell proliferation, was reported both *in vitro* and *in vivo* by the association of vemurafenib and the MEK inhibitors RO5068760 (51) or AS703026 (52), that increased Bim-E_L_ and the cleavage of caspase-3 and of its target poly(ADP-ribose) polymerase (PARP), along with a reduction of cyclin D1. A recent paper described the antitumor activity of the combination of TAK-733 (MEK inhibitor) and TAK-623 (pan-RAF inhibitor), able to impair cell proliferation, together with a reduction of pERK, pS6 and cyclin D1, and to enhance apoptosis by PARP cleavage (53). Inhibition of cell growth was also described by the association between U0126 (MEK1/2 inhibitor) with lonafarnib (a farnesyl transferase inhibitor, that reduces the post-translational activation of HRAS), but more interesting results came from the synergistic effects of lonafarnib with sorafenib (BAY 43-9006, a multikinase/RAF inhibitor that received FDA approval for the use in several solid tumor types). Indeed, besides significantly impairing BRAF^V600E^ and NRAS^Q61R^ melanoma cell growth and abrogating invasive tumor growth in organotypic skin culture, this combination not only induced upregulation of endoplasmic reticulum stress-related transcription factors p8 and CAAT/enhancer-binding protein (C/EBP) homologous protein (CHOP), but also significantly increased apoptosis (associated with the reduction of Bcl-2, Bcl-X_L_ and Mcl-1) (54). Finally, an unusual effect of simultaneous targeting of the RAS/RAF/MEK/ERK pathway molecules was described by Baudy *et al*, which observed that co-treatment of metastatic melanoma cells with cobimetinib (a MEK inhibitor also indicated as GDC-0973) and vemurafenib resulted in reduction of the fluorodeoxyglucose (FDG) uptake, associated with lower levels of glucose transporter (GLUT-1) at the cellular membrane of BRAF^V600E^ cells both sensitive and resistant to vemurafenib. This event was accompanied by the synergistic inhibition of CRAF, pMEK, kinase suppressor of RAS (Ksr), hypoxia-inducible factor 1-α (HIF-1α), the metabolic enzyme hexokinase II (Hxk II) and the transcription factor specificity protein 1 (Sp1), along with reduction of tumor volume *in vivo* (55).

These promising results obtained *in vitro* and in mouse models supported preliminary clinical studies that have given encouraging outcomes about the vertical targeting of MAPK pathway, as will be described in chapter 8.

### Parallel co-targeting of MAPK and PI3K pathways

Resistance to targeted therapies can arise from several mechanisms, including the hyper-activation of one or more pro-survival signaling pathways, like the PI3K/Akt/mTOR and RAS/RAF/MEK/ERK cascades that are known to interact at multiple points, resulting in cross-activation, cross-inhibition and pathway convergence (33). In 2008, Carracedo *et al* described for the first time the activation of the MAPK cascade, with increased pERK levels, after mTOR inhibition not only *in vitro*, but also in mouse models and in biopsies of breast cancer patients (56). Cooperation of these two main pro-survival pathways has been demonstrated in other tumor types such as prostate cancer (57), underlying the importance of a co-targeting approach in cancer therapy. Thus, a large set of combinatorial preclinical studies has been developed also in melanoma (Table III).

Selumetinib was combined with different PI3K/mTOR inhibitors, such as the PI3K inhibitor BEZ235 or the mTOR kinase blocker AZD8055, leading to *in vitro* synergistic reduction of cell viability, enhanced apoptosis (34,38), *in vivo* tumor regression and extension of median survival (58–60). Mechanistically, the modulation of several markers of proliferation (decreased pAkt and GSK3α/β) and apoptosis (increased Bim protein, cleavage of PARP and caspase-7 and reduced Mcl-1) was described (60). Furthermore, knockdown of Akt [by small interfering RNA (siRNA)] confirmed that the inhibition of the PI3K/Akt pathway sensitizes melanoma cells to selumetinib (60). Selumetinib has also been associated with other Akt/mTOR inhibitors (MK-2206, rapamycin or AKTi), as described by many groups that confirmed a reduction in cell viability, the blockade of cell cycle progression and concomitant enhanced apoptosis in different melanoma models (human, murine and canine) (34,45,61–63). Ambrosini *et al* showed how BRAF mutant uveal cells treated with selumetinib and MK-2206 died by apoptosis (associated with increased Bim and cleaved-PARP levels and decreased pBad expression), while cells harboring mutations in GNAQ oncogene underwent autophagia upon the same treatment (62). In addition, other MEK inhibitors (PD98059, U0126, cobimetinib, E6201, PD0325901 and trametinib) were used in several combinations with compounds that target the PI3K/Akt/mTOR pathway (LY294002, GDC-0941, rapamycin, GSK2126458 and the FDA approved temsirolimus), yielding interesting results in reducing tumor growth *in vivo* and *in vitro*, decreasing cell viability and concurrently improving apoptosis (associated with cleavage of PARP, increased caspase-3/7 activity and upregulation of Bim, together with downregulation of Bcl-2, Mcl-1, cIAP-2 and Apollon) (41,49,64–73). Among these combinations, the co-treatment with PD98059 or U0126 and LY294002 impaired migration, tumor cells invasion and tumor angiogenesis (64,74–76), associated with decrease of matrix metalloproteinase-2 (MMP2), inhibition of vascular-endothelial growth factor (VEGF) secretion and expression, and strong decrease of both basic fibroblast growth factor (bFGF) and HIF-1α (74–76). To support the anti-proliferative and pro-apoptotic effects of dual targeting MAPK and PI3K pathways, Posch *et al* described that different combinations of several inhibitors of MEK and PI3K/mTOR affected genes involved in cell division, in addition to inducing substantial decrease of cyclin D1 and upregulation not only of tumor suppressor genes but also of pro-apoptotic genes (72).

Several groups evaluated also the effect of combinatorial inhibition of RAF and PI3K/Akt/mTOR in improving antitumor response. Indeed, encouraging results were obtained in reduction of tumor cell proliferation and viability *in vitro*, and also in the impairment of invasive melanoma growth *in vivo*, combining vemurafenib with AZD8055 or BEZ235 (34) and sorafenib with the PI3K inhibitor wortmannin (64) or with LY294002 (41). Enhanced apoptotic cell death, associated with the modulation of its main mediators (such as cleavage of PARP, activation of caspase-3/7, enhancement of Bim protein levels and down-modulation of Bcl-2 and Mcl-1), and reduced pro-survival inputs (phosphorylated forms of Akt, S6, MEK and ERK), were induced by other combinations: co-treatment with dabrafenib and GSK2126458 (49); co-administration of PLX4720, rapamycin and the PI3K inhibitor PX-866 (77); PLX4720 with LY294002 (78); sorafenib with rapamycin (65,79); and vemurafenib with MK-2206 (51,80). A similar effect was shown by Boisvert-Adamo and Aplin: they bypassed the resistance conferred by hyper-activated MAPK and PI3K signaling pathways and enhanced apoptosis by treating BRAF-silenced melanoma cells with LY294002 (81). Interestingly, a triple treatment with dabrafenib, trametinib and GSK2126458 significantly reduced tumor growth compared to single treatments and to the association of dabrafenib and trametinib (82).

As shown by the above papers, the improvement of the anticancer efficacy obtained with the combined inhibition of MAPK and PI3K pathways, in respect to single treatment, is encouraging for clinical therapy perspective; indeed, several clinical trials of combinatorial treatment targeting both signaling cascades are ongoing, also on melanoma patients (for further details, see ClinicalTrials.gov).

## 4. Combination of RTKs targeting and other pro-apoptotic strategies

RTKs comprise several families of cell surface receptors that regulate critical cellular processes, including cell growth and survival. After ligand binding, receptors dimerize or oligomerize, with consequent autophosphorylation and substrate phosphorylation. Signaling through RTKs ultimately leads to the activation of two main pathways, MEK/ERK and PI3K/mTOR (28). Different RTKs and their downstream signaling pathways are aberrantly activated in melanoma cells and play a role in resistance to therapies (reviewed in ref. 28). Activating KIT mutations have been identified in some melanoma subtypes (mucosal, acral and non-sun-exposed), and FGF receptor (FGFR)-1 mutations have been sporadically found in melanoma (83–85). Furthermore, somatic mutations are present in genes coding for v-erb-b2 avian erythroblastic leukemia viral oncogene homolog (ErbB)4 (19%), EphB2, EphB6 and VEGF receptor (VEGFR)-1 (9–10% each), and with lower frequency in MET, TYRO3, EPHA2 and NTRK1–3 genes (86,87). Several RTKs are overexpressed or hyper-activated in melanoma compared to normal skin or benign nevi, including VEGFR-2, platelet-derived growth factor receptors (PDGFRs), FGFRs, epidermal growth factor receptor (EGFR) and ErbB3 (88–93). Increased RTK activity can be also induced in melanoma cells by aberrant autocrine or stromal secretion of RTK ligands, such as VEGF, FGF, hepatocyte growth factor (HGF) and insulin-like growth factor-1 (IGF-1) (29,94,95); interestingly, increased VEGF, FGF or HGF concentration in patients’ sera or neoplastic tissue correlated with poor prognosis (29,94).

Two compounds acting on multiple families of RTKs have shown anti-melanoma pre-clinical activity in combinatorial therapies *in vitro* (Table IV for all pre-clinical studies combining RTKs targeting and other pro-apoptotic strategies). Sunitinib is a multi-targeted tyrosine kinase inhibitor that potently inhibits VEGF, PDGF and c-kit receptor kinases (96), and was approved by the FDA for the treatment of renal cell carcinoma and gastrointestinal stromal tumor. The proteasome inhibitor bortezomib (Velcade/PS-341) is approved by FDA for treating relapsed multiple myeloma and mantle cell lymphoma. It induced growth arrest of melanoma cells (but not of normal melanocytes) both *in vitro* and in a murine xenograft model (97,98), but a phase II clinical trial using single-agent bortezomib in patients with advanced melanoma yielded disappointing results (99). Interestingly, in sunitinib-sensitive melanoma cell lines, the association of sunitinib with bortezomib resulted in synergistic decrease of cell viability, increase in caspase-3 activation and apoptosis (100). Dasatinib is another compound that targets multiple RTKs, in addition to Src kinases (101,102); it has been approved by FDA for first line use in patients with chronic myelogenous leukemia (CML) and Philadelphia chromosome-positive acute lymphoblastic leukemia (Ph^+^ ALL). Combination of dasatinib with U0126 increased intra-spheroid cell death and reversed MEK-induced invasive phenotype of melanoma cell lines (103).

Specific blockade of different RTK families also cooperates with targeted therapy, in agreement with the hyper-activation of several RTKs in melanoma. Hamai *et al* demonstrated that c-kit inhibition through imatinib (FDA-approved as first-line treatment for Ph^+^ CML) increased TRAIL-mediated proliferation impairment and apoptosis in a primary TRAIL-sensitive melanoma cell line but not in its metastatic TRAIL-resistant counterpart. In this model, imatinib did not increase TRAIL receptor expression on cell surface, excluding the involvement of such mechanism; sensitization to TRAIL by imatinib was instead associated with accelerated crosstalk between the extrinsic and intrinsic apoptosis pathways mediated by downregulation of the anti-apoptotic factor FADD-like IL-1β-converting enzyme (FLICE)-inhibitory protein (cFLIP_L_), increased Bax:Bcl-X_L_ ratio and Bax:Bcl-X_L_/Bcl-2 translocation, thus inducing cytochrome c release and ultimately activation of caspase-8, -9 and -3 (104). The combination of imatinib and the anti-VEGFRs vatalanib (but not single treatments) improved the therapeutic efficacy of chemotherapy with paclitaxel in reducing the growth of mouse B16 tumor stably expressing PDGF-BB; a similar but less pronounced effect was obtained with siRNA-mediated c-kit downregulation, indicating a role of c-kit in resistance to paclitaxel (105).

The enhanced activity of ERK and PI3K/Akt pathways consequent to RTKs aberrant activation could account for resistance to therapies targeting these cascades; this provides the rationale for combining ERK and/or PI3K blockade with strategies leading to the inhibition of different families of RTKs.

Confirming the involvement of VEGF in melanoma development, the anti-VEGF monoclonal antibody (mAb) bevacizumab (FDA-approved for various metastatic cancers) inhibited the proliferation of VEGFR-2^+^ (but not of VEGFR-2^neg^) cell lines, but did not cause cell death; survival was at least in part mediated by mTOR pathway, as co-treatment with bevacizumab and rapamycin caused VEGFR-2-dependent loss of half of the VEGFR-2^+^ cells (106). Accordingly, the association of everolimus and vatalanib had synergistic effect in reducing tumor growth and lymph node metastases in B16 mouse model, which was associated with reduction of plasma VEGF (107). Furthermore, combined inhibition of VEGF (with bevacizumab) and the other RTK EGFR (with erlotinib) in a severe combined immunodeficient mouse xenotransplantation model led to a synergistic reduction in tumor volume, and this combination was effective also in decreasing the number of lymph node and lung metastases; this activity was associated with erlotinib-mediated impaired cell proliferation and synergistically increased apoptosis and reduced tumor angiogenesis (108).

Straussman *et al* demonstrated the importance of stromal-derived mediators in melanoma resistance to PLX4720; the factor which correlated best with proliferation rescue was the MET ligand HGF, whose binding to MET resulted in activation of the MAPK and PI3K/Akt signaling pathways and resistance to MEK/RAF blocking (29). Indeed, dual blockade of MEK/BRAF^V600E^ (with PD184352, PLX4720 or vemurafenib) and MET (with crizotinib, SU11274 or MET-specific siRNA) resulted in reversal of drug resistance and reduced the proliferation of melanoma cell lines, which was restored by exogenous HGF (29,109). Interestingly, both vemurafenib and crizotinib have already received FDA approval for melanoma and some forms of non-small cell lung cancer, respectively.

Synergistic reduction in cellular viability and increase in apoptosis of melanoma cell lines were obtained by co-treatment with FGFR inhibitors (PD166866 or SU5402) and vemurafenib or the multikinase/RAF inhibitor sorafenib, and were associated with reduced expression of the phosphorylated forms of ERK, Akt and Stat3; furthermore, sorafenib enhances the *in vivo* antitumor effect of FGF signal blockade with dominant-negative receptor constructs (90).

The important role of ErbB family and IGF-1R in melanoma growth and resistance to targeted therapy has been reported. Treatment with the two FDA-approved compounds gefitinib (EGFR inhibitor) and lapatinib (ErbB2/EGFR inhibitor), or with anti-ErbB3 mAb can overcome ErbB-mediated resistance to BRAF or MEK inhibitors (PLX4720 or single/combined vemurafenib and trametinib), diminishing melanoma cells growth both *in vitro* and *in vivo* (110–112); this effect was associated with synergistically reduced pAkt and pERK (111,112). IGF-1R blocking by cyclolignan picropodophyllin (PPP) increased TRAIL-induced apoptosis of both TRAIL-sensitive and TRAIL-resistant melanoma cell lines (113); moreover, combined treatment with PPP and trametinib induced apoptosis of BRAF-inhibitor resistant cells, which was associated with trametinib-dependent reduction of pERK and pBAD, PPP-dependent decreased pAkt and synergistically reduced Mcl-1 (114).

Collectively, the above papers confirm the role of RTKs in melanoma cells survival and resistance to the most commonly used targeted therapy strategies, thus suggesting the potential clinical benefit of combinations involving RTKs blockade and other selective inhibitors. Since many papers showed some heterogeneity in the expression of different RTKs and their ligands by melanoma cells (29,90,100,106,111), a pre-therapy evaluation of their levels in the tumor should be recommended.

## 5. Association of death receptor engagement and signaling pathway inhibition

Several pre-clinical studies have investigated the possibility to directly induce tumor cell death through the engagement of death receptors, which are expressed at various degrees by melanoma cells (115,116). Moreover, agonistic antibodies or death receptor ligands induce apoptosis independently of the p53 status and may therefore overcome the p53-dependent melanoma resistance to cell death (117).

Clinical studies with compounds targeting the death receptor family were initiated several years ago in tumors other than melanoma, with the use of recombinant human (rh)TNF-α. Unfortunately, only in rare cases clinical activity was seen and severe adverse events have been a major hurdle, and to date TNF-α in melanoma is only used as a ‘facilitator’ to increase penetration of chemotherapeutic agents in the settings of single limb perfusion of in-transit metastases (118). Toxicity towards hepatocytes and non-transformed cells was seen also for treatments with Fas ligand/CD95L; nevertheless, a novel preparation is being tested for safety and activity on different tumor types (119).

Among the death receptor ligands, APO2L/TRAIL has been shown to induce apoptosis in a variety of transformed cells while sparing normal cells, and constitutive expression of TRAIL receptors has been observed in a variety of tumor types. TRAIL receptors engagement has therefore been identified as a promising strategy for cancer treatment (120). Moreover, TRAIL receptor-agonistic antibodies or soluble TRAIL (dulanermin) was well tolerated in phase I/II studies on different malignances, but revealed limited efficacy as single agent, likely due to intrinsic and inducible resistance (121). Indeed, different combinatorial strategies have been investigated to overcome intrinsic or acquired melanoma resistance to TRAIL-induced apoptosis, mainly through the targeted blockade of proteins involved in different signaling pathways as well as survival, transcription or metabolism (Table V).

One of the most promising approaches to sensitize melanoma cells to TRAIL-mediated cell death is the simultaneous targeting with inhibitors of either MEK/ERK or PI3K/mTOR pathways. The pre-treatment of human melanoma cell lines with U0126 demonstrated the crucial role of ERK1/2 activation in protecting tumor cells from rhTRAIL-induced apoptosis. This association enhanced cell death through increased mitochondrial depolarization and Smac/DIABLO release, associated with relocation of Bax from the cytosol to mitochondria, augmented caspase-3 and -9 activation, and reduction in XIAP levels (122). A similar mechanism was pointed out for the pan-RAF inhibitor L-779,450: co-treatment with soluble TRAIL has been recently shown to overcome TRAIL resistance of both BRAF-mutant and BRAF^wt^ melanoma cells by increasing apoptosis through release of cytochrome c and Smac/DIABLO from mitochondria and augmented activation of Bax and Bim. The synergy in cell death induction was shown also for combination of TRAIL and U0126 or vemurafenib (123).

Moreover, wortmannin or MK-2206 have been shown to enhance the apoptotic cascade activation and the consequent death after TRAIL treatment of melanoma cell lines, selected for different susceptibility profiles to soluble TRAIL, through a mechanism involving increased reactive oxygen species (ROS) production and augmented Bax activation via phosphorilation of threonine-167 (124).

Furthermore, the targeting of cell adhesion and motility through the inhibition of Src kinase (with either PP2 or AZD0530), PI3K (with PI103) or MEK (with U0126) has been shown to cooperate with TRAIL to induce a reduction in cell viability of TRAIL-resistant 1205Lu melanoma cells (125).

Several preclinical studies have investigated the potential synergy between TRAIL and HDAC inhibitors, a class of compounds that block HDACs from removing acetyl groups from histone tails, thereby preventing the silencing of pro-apoptotic genes and regulating the expression of non-histone proteins (i.e., apoptosis-associated genes) (126). The HDAC inhibitor suberoyl bis-hydroxamic acid (SBHA) synergized with soluble TRAIL *in vitro* to induce mitochondrial-mediated melanoma cell death (127,128), and similar results in terms of cell growth arrest and caspase-dependent apoptosis were obtained with full-length TRAIL, delivered from an adenoviral vector, in combination with the HDAC inhibitor vorinostat (suberoylanilide hydroxamic acid (SAHA)) (129). The increased apoptosis correlated with enhanced mitochondrial apoptotic events and increased activation of pro-apoptotic proteins like Bax, Bak, Bim and Bid, whereas the expression of Mcl-1, XIAP and Bcl-X_L_ was reduced (127–129). Additive effects in terms of apoptosis induction were observed also for the combination of TRAIL and the calcineurin/nuclear factor of activated T cells c2 (NFATc2) inhibitor AM404 (130).

In a recent study, Zimmerman *et al* showed that activation of Wnt/β-catenin signaling increased TRAIL-induced apoptosis in melanoma cells: A375 cells treated with CHIR99021 (GSK-3 inhibitor) exhibited a dose-dependent increase in the percentage of cells undergoing apoptosis upon rhTRAIL treatment compared to control treated cells (with AXIN-1 playing a major role in apoptosis-sensitization) (131). An efficient sensitization for TRAIL-induced apoptosis of either TRAIL resistant or TRAIL sensitive melanoma cell lines was also achieved by the combination with BMS-345541 [IκB kinase (IKKβ) inhibitor]; the synergistic effects can be explained by the activation of Bid by TRAIL and of Bax by BMS-345541. The critical roles of XIAP, Smac and Bid in the synergistic interactions were proven (132).

Several papers showed how interfering with the expression of one or more molecules involved at different levels of either the extrinsic or the intrinsic apoptosis cascade can dramatically enhance melanoma susceptibility to TRAIL. Normally, the activity of the IAP family member XIAP is antagonized by Smac/DIABLO, which is released from mitochondria in response to apoptotic stimuli. Indeed, compounds mimicking this tetrapeptide, called Smac mimetics, have shown to powerfully potentiate TRAIL-induced caspase-dependent apoptosis in melanoma cells *in vitro*, and to an even higher extent if combined with bortezomib (133). Furthermore, a strong synergy between a proteasome inhibitor (in this case NPI-0052 or DHMEQ) and TRAIL in induction of melanoma cell death has been reported also by Baritaki *et al*; the effect probably involved a reduction in NF-κB signaling and in levels of Snail and RKIP (134). Remarkably, siRNA for several IAPs (cIAP-1, cIAP-2, XIAP, Apollon, Livin and Survivin) or for Bcl-2 have been used not only *in vitro* studies with soluble or membrane-bound TRAIL (68,135), but also *in vivo* in combination with AdhCMV-TRAIL, obtaining increased cell death and reduced tumor volume (136), confirming the importance of these molecules in melanoma resistance to TRAIL-dependent cell death. Similarly, a siRNA specific for Mcl-1 was proven to be able to sensitize melanoma cells to anti-Fas mAb-induced melanoma apoptosis (137); and siRNAs for the anti-apoptotic TRAIL-resistance factor cFLIP (138) have been shown to enhance melanoma apoptosis after treatment with either soluble TRAIL or Fas ligand through the increment of caspase-8 activity (139).

The results presented in this section demonstrate how the triggering of death receptors in combination with the inhibition of different pro-survival processes effectively increases melanoma cell death, and therefore might be a potentially effective antitumor strategy for melanoma treatment. Overall, the best results were obtained when TRAIL was associated with inhibitors of survival pathways hyper-activated in melanoma, suggesting that these strategies deserve to be evaluated for the design of future clinical trials.

## 6. Combined blockade of anti-apoptotic proteins and MAPK cascade

Increased expression or activation of several anti-apoptotic molecules is a common feature of melanoma cells, leading to resistance to death-inducing treatments (140). Specifically, inhibitors of MAPK pathway trigger the apoptosis cascade, but are insufficient to kill melanoma cells, revealing an intrinsic resistance; this might be mediated by low levels of BH3-only proteins and/or high levels of Bcl-2-like prosurvival proteins (12,13).

To overcome this hurdle, several groups evaluated the association of ABT-737 [a BH3-mimetic that inhibits Bcl-2, Bcl-X_L_ and Bcl-w and shows promise for treating leukemia, lymphoma and small-cell lung cancer (141,142)] with blockers of MEK (U0126, PD0325901, PD98059, CI-104) or BRAF^V600E^ (PLX4720) (see Table VI for all the preclinical studies co-targeting anti-apoptotic proteins and MAPK pathway). These combinations induced higher level of caspase-dependent apoptosis than single agent; this effect was mediated by MEK or BRAF inhibition-dependent translocation of Bmf or upregulation of Bim, which led to an increased sequestration of Mcl-1, and by the cleavage of caspase-9, -3 and of PARP (12,143,144). Furthermore, the association of PD0325901 with ABT-737 inhibited tumor growth and induced tumor regression *in vivo* (12). Interestingly, while the combined treatment with PLX4720 and ABT-737 induced apoptosis in melanoma cell lines established from pre-therapy lesions, this effect was lost in cell lines from post-therapy lesions of patients who developed resistance to the BRAF inhibitor (143). Furthermore, the association of selumetinib with ABT-263, a BH3-mimetic that disrupts the interactions of Bcl-2 and Bcl-X_L_ with pro-apoptotic proteins (145), also induced caspase-dependent cell death in A375 melanoma cell line (146).

Because no effective synthetic inhibitor of Mcl-1 had been previously described, Verhaegen *et al* used a computational approach to generate TW-37, the first rationally designed BH3 mimetic able to block Mcl-1, Bcl-X_L_ and Bcl-2. TW-37 synergized with MEK inhibitors (U0126 or CI-1040) in inducing apoptosis and activation of initiator and effector caspases, although the cell death was not only caspase-dependent. Interestingly, this association had minimal toxicity against normal skin cells. The combinatorial treatment increased the release of cytochrome c and Smac/DIABLO compared to single agents, and induced activation of Bax/Bak, production of ROS and p53 activity. CI-1040 plus TW-37 also blocked tumor growth in xenografted mice (147).

It has been recently shown that Bcl2-A1 is highly expressed in melanoma cell lines and can confer resistance to BRAF inhibition, since its knockdown increased sensitivity to PLX4720 (148). ABT-737 has no effect on Bcl2-A1, therefore PLX4720 was associated with obatoclax, which inhibits the anti-apoptotic Bcl-2-family members, including Bcl2-A1. This combination synergistically induced apoptosis in a Bcl2-A1 amplified cell line, and it decreased tumor volume *in vivo* without significant toxicity (149).

p38 MAPK pathway is strongly activated in cells in response to stress signals, growth factors and inflammatory cytokines, and controls cell survival, migration and invasion (150). Keuling *et al* showed that also the combination of ABT-737 and p38 inhibitors (SB202190 or SB203580) synergistically induce apoptosis and the activation of caspase-8, -9, -3 in melanoma cell lines. These effects were most prominent with a triple combinatorial treatment with Mcl-1 knockdown by siRNA. Interestingly, therapy with either ABT-737 plus SB202190 or plus Mcl-1 siRNA promoted caspase-dependent cleavage of different Puma isoforms, possibly involved in the response to treatment (151).

Recently, we found that Apollon was expressed in melanoma cells, and that cell death in response to PLX4720 or PD0325901 correlated with Apollon downmodulation. Silencing of Apollon by siRNA increased apoptosis in response to the above-mentioned drugs, irrespective of their p53 status. Mechanistically, Apollon siRNA enhanced Bcl-x downregulation, Bax and Bad upregulation, mitochondrial depolarization and caspase-8, -9, -2 and -3 activation (68).

The studies presented above confirmed the crucial role of anti-apoptotic molecules such as Bim, Bmf, Bax, Bak, anti-apoptotic Bcl-2 family proteins and several IAPs in controlling melanoma sensitivity to inhibitors of the MAPK pathway. Accordingly, the association of widely used inhibitors, like U0126, PLX4720 or selumetinib, to compounds that specifically target apoptosis-related proteins showed promising antitumor activity.

## 7. Co-targeting of proteasome, HDAC, anti-apoptotic molecules and/or survival pathways

Besides the cellular processes discussed above, in recent years several other molecules have been described as essential for melanoma survival and therefore potential therapeutic targets; these include proteasome, HDAC and Stat3 (97,98,152,153). Consequently, pre-clinical melanoma models have been used to test different additional combinations of targeted therapies (Table VII).

Following bortezomib treatment, melanoma cells upregulate anti-apoptotic Bcl-2-family proteins, especially Mcl-1 (154,155); accordingly, the association of bortezomib with various strategies aimed either at specifically reducing the activity of these proteins [Mcl-1 siRNA, (−)gossypol or obatoclax] or at promoting apoptosis (the peroxisome proliferator activated receptor (PPAR)-γ agonist rosiglitazone or Smac mimetics) synergistically reduced cell viability and induced cell death *in vitro*, as well as inhibited tumor growth and lung metastasis development in a melanoma xenograft model (133,154–157). When the mechanism was investigated, the increased apoptosis was found to be mediated by cleavage of PARP and of caspase-8, -9, -7 and -3, and by Bak and Bax activation (133,154,155). In the case of (−)gossypol, *in vitro* activity was dependent on Mcl-1 and to a lesser extent on Bcl-X_L_, but not on Bcl-2 (155). Accordingly, MG-132 or bortezomib combined with ABT-737 led to enhanced reduction in cell viability, induction of Noxa-dependent apoptosis mediated by activation of Bak/Bax and of caspase-3, and decreased tumor growth *in vivo* (158,159). Further co-treatments showed that melanoma resistance to proteasome inhibitors might be also mediated by different pathways: the combination of bortezomib with LY294002 synergistically diminished cell growth and increased sub-G0/G1 fraction and cleaved caspase-3 (100); additionally, the association of BSc2118 with the calpain inhibitor PD150606 reduced viability of cisplatin-resistant cells compared to single treatments, suggesting that the protease calpain might play a role in chemotherapy- and proteasome inhibitor-resistance in melanoma cells (160); finally, co-treatment with marizomib and the HDAC inhibitor vorinostat synergistically impaired cell growth and increased sub-G0/G1 fraction (161). The association of the same HDAC inhibitor and PLX4720 or selumetinib was effective in inducing proliferation arrest and cell death *in vitro* and in reducing tumor growth *in vivo*. This effect was associated with de-repression of Bim-E_L_ expression, activation of PARP, caspase-9 and -3 and release from mitochondria of cytochrome c and Smac/DIABLO; despite this, at least in some cell lines Bim and caspase-3 were dispensable for cell death mediated by the combination of vorinostat and PLX4720, which induced cell necrosis (46,162).

Furthermore, sensitivity of MEK-inhibitor-resistant melanoma cell lines to MEK/ERK blockade could be restored by inhibition of Stat3, which is constitutively activated in human melanoma and contributes to cell growth and survival (163). Indeed, Stat3 is activated by phosphorylation in melanoma cells that acquired an invasive phenotype following treatment with U0126 or with the BRAF-inhibitor SB590885, and Stat3 knockdown or its inhibition with CPA-7 prevented the appearance of an invasive phenotype and increased cell death in the presence of U0126 or SB590885 (103).

Recent papers also showed that melanoma cell death might be potentiated by the co-inhibition of two anti-apoptotic molecules. Unfortunately, melanoma cells are insensitive to single ABT-737 treatment, and Mcl-1 might have a major role in this resistance (164,165). Accordingly, the association of ABT-737 with specific Mcl-1 targeting (with siRNA or maritoclax) or with the Bcl-2-family inhibitor obatoclax, along with Noxa overexpression, has produced interesting results in terms of reduction in melanoma cell viability and colony formation and of apoptosis induction; these effects were associated with increased Bim expression, Bid and Bax activation, cleavage of PARP and of caspase-9, -8, -3 (148,156,166–168).

Collectively, these results suggest that effective anti-melanoma therapy might include strategies directed at blocking key molecular events such as proteasome activity, HDAC and Stat3; their targeting might be effectively associated with the inhibition of either hyper-activated pathways or anti-apoptotic molecules, in order to achieve better disease control. Furthermore, even the combined blockade of two anti-apoptotic proteins has shown interesting pre-clinical activity, thus suggesting that also this strategy might be exploited for possible clinical application.

## 8. Clinical trials using combinatorial targeted therapy

In recent years, several clinical trials have been conducted in melanoma patients to test the efficacy of the combinatorial approaches that gave promising results in the pre-clinical setting (Table VIII). The studies conducted so far with the aim of evaluating the safety and efficacy of combined MEK- and mTOR/PI3K-inhibition combination obtained disappointing results. A phase I trial of dose escalation of sorafenib (orally once or twice daily) and temsirolimus [intravenously (i.v.) weekly] was conducted on 25 stage IV or unresectable or recurrent stage III melanoma patients; the maximum tolerated doses were sorafenib 400 mg every morning and 200 mg every evening and temsirolimus 25 mg weekly, but this trial failed to achieve any clinical response (no complete responses (CRs) or partial responses (PRs), 10 patients achieved stabilization of disease as their best response) and the median PFS was 2.1 months (169). A further randomized phase II study of sorafenib (200 mg twice daily) plus temsirolimus (25 mg weekly) or sorafenib (400 mg every morning and 200 mg every night daily) plus the farnesyltransferase inhibitor tipifarnib (orally 100 mg twice daily, 3 weeks of every 4) was conducted in patients with non-ocular melanoma, no prior systemic chemotherapy and no history of brain metastasis, but these combinations did not show sufficient activity to justify further use: among the 63 patients treated with sorafenib and temsirolimus, the median progression-free survival (PFS) was 2.1 months and median OS was 7 months; 39 evaluable patients were accrued to sorafenib plus tipifarnib arm, which closed after first-stage accrual having reached a median PFS of 1.8 months and an OS of 7 months, with 1 patient (3%) achieving PR (170). This last part was in contrast with a previous phase I trial of oral sorafenib (400 mg once or twice daily or 400 mg every morning and 200 mg every evening) plus tipifarnib (100–300 mg orally daily, 3 weeks of every 4) in patients with different advanced malignancies, which indicated possible clinical benefit in melanoma patients [3/7 (43%) had stable disease (SD) of 4, 4 and 14 months] (171).

More encouraging results were derived from a phase I–II trial evaluating the vertical targeting of MAPK pathway, by the association of oral dabrafenib and trametinib at escalating doses. The study involved 247 patients with metastatic melanoma and BRAF^V600^ mutations; the maximum tolerated dose combination was not reached in this study, and the recommended phase II dose was the highest phase I dose level (150 mg of dabrafenib twice daily plus 2 mg of trametinib once daily). The patients receiving this combination had improved median PFS (9.4 vs. 5.8 months) and response ratio (76 vs. 54% CR or PR, 10.5 vs. 5.6 months the median duration of response) compared with patients receiving dabrafenib monotherapy (172). The emergence of cutaneous squamous cell carcinoma early in the course of BRAF-inhibitor therapy has been associated with paradoxical MAPK pathway activation during BRAF inhibition (173); interestingly, the incidence of cutaneous squamous-cell carcinoma in patients receiving the phase II dose of dabrafenib and trametinib combination was 7%, compared with 19% after dabrafenib monotherapy (172).

Promising results came also from studies in which RTK antagonists were combined with the inhibition of other pathways. Two phase II trials showed that the association of i.v. bevacizumab with the mTOR inhibitors everolimus or temsirolimus in metastatic melanoma patients is generally well tolerated and has moderate clinical activity: after bevacizumab (15 mg/kg every 21 days) plus everolimus (10 mg orally daily) in metastatic melanoma patients who had received up to 2 previous systemic regimens (chemotherapy and/or immunotherapy, no previous treatment with angiogenesis or mTOR inhibitors), 7/57 patients (12%) achieved major responses (1 CR and 6 PR), with 33 (58%) SD at 6 weeks; the median PFS and OS were 4 and 8.6 months, respectively. The objective response rates were similar in previously untreated patients compared with those who had received 1 or 2 previous regimens (174). Another phase II trial associated bevacizumab [(10 mg) every 2 weeks] with temsirolimus (25 mg i.v. weekly) in 17 unresectable stage III–IV melanoma patients, of which 3 (18%) experienced PR and 9 (53%) had SD at 8 weeks. Among 10 evaluable patients with BRAF^WT^ tumors, 3 had PR (33%), 5 had SD (50%) and 2 had PD (20%), indicating that the clinical activity of bevacizumab plus temsirolimus might be greater in these patients (175). Also the association between sorafenib (400 mg orally twice daily) and the MET inhibitor tivantinib (ARQ 197, 360 mg orally twice daily) in a phase I study on 16 melanoma patients was well tolerated and exhibited preliminary anticancer activity (6% CR, 19% PR and 19% SD, 5.3 months the median PFS). Interestingly, among 8 patients with NRAS mutations, median PFS was 9.2 months and responses were 1 CR (13%), 1 PR (13%) and 2 SD (25%) (Means-Powell JA, *et al*, J Clin Oncol 30: 15, abs. 8519, 2012).

Furthermore, the heat shock protein 90 inhibitor tanespimycin (300–450 mg/m^2^ i.v. on days 1, 8 and 15 in a 28-day cycle) has been associated with sorafenib (400 mg orally twice daily, starting at day 14 before tanespimycin) in patients with metastatic or unresectable solid malignancies. This phase I study concluded that the recommended phase II doses of this combination are 400 mg sorafenib twice daily and 400 mg/m^2^ tanespimycin on days 1, 8 and 15, every 28 days, and clinical efficacy was observed in melanoma patients [4 of 6 (67%) showed SD, with mean duration of 3.4 months] (176).

Finally, the combined inhibition of the proteasome (with marizomib, 0.15–0.7 mg/m^2^ i.v. on days 1, 8 and 15 of 28 day cycles) and of HDAC (with vorinostat, 300 mg orally every day on days 1–16 of each cycle) in patients with solid tumors (with the exclusion of patients with brain metastases and prior treatment with other HDAC- or proteasome-inhibitors) has been shown in a phase I trial to be well tolerated, with indications of anti-melanoma activity [SD in 11/14 (79%) melanoma patients, of which 4 maintained SD for at least 4 months] (161).

## 9. Conclusions

Melanoma development, survival, progression and chemoresistance are regulated by several heterogeneous factors. Due to this complexity, tumor cells are susceptible to different types of targeted therapy, but unfortunately single agents often have limited efficacy due to intrinsic or acquired resistance mechanisms. The improved elucidation of the role of different molecular processes in such resistance paved the way for combinatorial therapies, which gave promising results in pre-clinical and in some clinical studies. Importantly, many of the drugs used in the pre-clinical studies presented in this review have already been used in clinical practice, either as single agents or in tumors other than melanoma, thus rendering more potentially feasible the clinical validation of such combinations. Interestingly, some pre-clinical studies have also shown off-target effects on the immune system and on the different steps of antitumor immune response following therapy with many of selective inhibitors discussed above (177). Indeed, as immunomodulating agents such as cytotoxic T lymphocyte associated antigen-4 and programmed death-1 inhibitors have demonstrated clinical activity in melanoma (178,179), their association with selected targeted therapy strategies might lead to improved antitumor efficacy and ultimately to better clinical results.

## Figures and Tables

**Table I:** Preclinical studies evaluating vertical targeting of PI3K/Akt/mTOR pathway.

Compound 1 (target 1)	Compound 2 (target 2)	Effect of combination vs. single treatment	Mechanism of synergy	Refs.
Rapamycin (mTOR)	LY294002 or BKM-120 (PI3K)	↓ cell viability		(38)
Rapamycin (mTOR)	LY294002 (PI3K)	↓ cell proliferation, ↑ cell cycle arrest	↓ cyclin D1, pAkt, pS6K1 and p4EBP1	(39,41)
Rapamycin (mTOR)	ZSTK474 (PI3K)	↓ cell proliferation		(40)
Rapamycin (mTOR)	PI-103 (PI3K)	↓ cell viability, ↑ cell cycle arrest, ↑ apoptosis, ↓ tumor growth	↓ pAkt, pS6	(42)
Rapamycin (mTOR)	GDC-0941 (PI3K p110α)	↓ cell viability	↓pAkt, pS6	(42)

4EBP, eukaryotic translation initiation factor 4E binding protein 1; Akt, v-Akt murine thymoma viral oncogene; mTOR, mammalian target of rapamycin; PI3K, phosphatidylinositol-4,5-bisphosphate 3-kinase; S6K1, ribosomal protein S6 kinase 1.

**Table II:** Preclinical studies evaluating vertical targeting of RAS/MEK/ERK pathway.

Compound 1 (target 1)	Compound 2 (target 2)	Effect of combination vs. single treatment	Mechanism of synergy	Refs.
Selumetinib (MEK1/2)	PLX4720 (BRAF^V600E^)	↓ cell viability and cell growth	↓ pERK	(44–46)
Trametinib (MEK1/2)	Dabrafenib (BRAF)	↓ cell proliferation and viability, ↓ skin lesions, ↓ tumor growth	↓ pERK, cyclin D1 and pRb, ↑ p27, ↓ CCND1, CDC25A, PCNA, MYC, MCL1 mRNA, ↑ BIK and CARD6 mRNA	(47–49)
Trametinib (MEK1/2)	PLX4720 (BRAF^V600E^)	↓ tumor growth, ↓ MEK inhibitor-associated skin toxicity		(50)
RO5068760 (MEK)	Vemurafenib (BRAF^V600E^)	↓ cell proliferation, ↓ cell cycle progression, ↑ apoptosis, ↓ tumor growth	↓ pERK and cyclin D1, ↑ Bim-E_L_ and cleaved-PARP,	(51)
AS703026 (MEK)	Vemurafenib (BRAF^V600E^)	↓ cell viability, ↑ apoptosis	↓ pERK, ↑ cleaved-caspase-3	(52)
TAK-733 (MEK)	TAK-623 (pan-RAF)	↓ cell proliferation, ↑ apoptosis	↓ pERK, pS6 and cyclin D1, ↑ cleaved-PARP	(53)
Sorafenib (multikinase/RAF) or U0126 (MEK1/2)	Lonafarnib (farnesyl transferase)	↓ cell growth, ↑ apoptosis, ↓ invasion	↓ Bcl-2, Bcl-X_L_ and Mcl-1, ↑ p8 and CHOP	(54)
Cobimetinib (MEK)	Vemurafenib (BRAF^V600E^)	↓ FDG uptake, ↓ tumor volume	↓ GLUT-1, CRAF, pMEK, Ksr, HIF-1α, Hxk II and Sp1	(55)

Bcl-2, B-cell lymphoma-2; CHOP, CAAT/enhancer-binding protein (C/EBP) homologous protein; ERK, extracellular signal-regulated kinase; FDG, fluorodeoxyglucose; GLUT-1, glucose transporter; HIF-1α, hypoxia-inducible factor 1-α; Hxk II, hexokinase II; Ksr, kinase suppressor of RAS; MEK, mitogen-activated protein/extracellular signal-regulated kinase; PARP, poly(ADP-ribose) polymerase; Rb, retinoblastoma; Sp1, specificity protein 1.

**Table III:** Preclinical studies co-targeting RAS/MEK/ERK and PI3K/Akt/mTOR pathways.

Compound 1 (target 1)	Compound 2 (target 2)	Effect of combination vs. single treatment	Mechanism of synergy	Refs.
Selumetinib (MEK1/2)	BEZ235 (PI3K)	↑ tumor regression and median survival, ↓ cell viability, ↑ apoptosis		(34,38,58)
Selumetinib (MEK1/2)	AZD8055 (mTOR)	↓ cell viability, ↑ apoptosis, ↑ tumor regression	↓ pAkt, GSK3α/β and Mcl-1, ↑ p27, Bim and cleaved-PARP and caspase-7	(59,60)
Selumetinib (MEK1/2) or PLX4720 (BRAF^V600E^)	MK-2206 (Akt)	↓ cell viability, ↑ apoptosis, ↓ tumor volume	↓ cyclin D1, ↑ Bim and cleaved-PARP, ↓ pBad	(45,62)
Selumetinib (MEK1/2) or vemurafenib (BRAF^V600E^)	AKTi or rapamycin (mTOR)	↓ cell viability, ↑ apoptosis	↓ pAkt, pS6 and p4EBP1, ↑ cleaved-caspase-3	(61)
Selumetinib (MEK1/2)	Rapamycin (mTOR)	↓ cell growth, ↑ G1 arrest		(63)
Dabrafenib (BRAF) or trametinib (MEK1/2)	GSK2126458 (mTOR/PI3K)	↓ cell growth, ↑ apoptosis	↑ cleaved-PARP, Bim and caspase-3/7 activity	(49,70)
Vemurafenib (BRAF^V600E^)	AZD8055 (mTOR) or BEZ235 (PI3K)	↓ cell viability, ↑ apoptosis		(34)
Selumetinib (MEK1/2)	AKTi	↓ cell viability	↓ pERK and pAkt	(34)
Sorafenib (multikinase/RAF) or PD98059 (MEK)	Wortmannin (PI3K) or LY294002 (PI3K)	↓ cell growth, ↑ apoptosis, ↓ migration and invasion		(64)
U0126 (MEK1/2)	LY294002 (PI3K)	↓ cell proliferation and viability, ↑ apoptosis, ↓ tumor incidence and growth, ↓ migration, invasion and angiogenesis	↓ MMP2, VEGF, cyclin D1, HIF-1α and bFGF, ↑ cleaved-caspase-3	(41,64, 74–76)
U0126 (MEK1/2) or sorafenib (multikinase/RAF)	LY294002 (PI3K)	↓ cell proliferation		(41)
U0126 (MEK1/2) or PD98059 (MEK)	Rapamycin (mTOR)	↓ cell proliferation		(41,65)
Sorafenib (multikinase/RAF)	Rapamycin (mTOR)	↓ cell proliferation, ↑ apoptosis, ↓ invasive growth	↓ Bcl-2 and Mcl-1	(65,79)
Dabrafenib (BRAF) and trametinib (MEK1/2)	GSK2126458 (mTOR/PI3K)	↓ tumor growth		(82)
Cobimetinib (MEK)	GDC-0941 (PI3K)	↓ tumor growth, cell viability and apoptosis	↑ Bim, cyclin D1 and cleaved-PARP, ↓ pS6	(67,71)
E6201 (MEK1)	LY294002 (PI3K)	↓ cell viability		(69,78)
JTP-74057 or PD325901 (MEK)	GSK2126458, BEZ235, PP242, GDC-0941 or GSK690693 (PI3K/mTOR)	↓ cell viability, ↑ apoptosis, ↑ tumor regression	↓ pERK, pAkt and pS6, ↑ caspase-3/7 activity, ↓ cyclin D1, ↑ RB1CC1 and STK11 mRNA, ↑ CABC1, MAP3K10, DAPK3 and MAP3K9 mRNA	(72)
PD0325901 (MEK)	Temsirolimus or rapamycin (mTOR)	↓ cell viability and tumor size, ↑ apoptosis	↓ pERK, p4EBP1, Apollon, cIAP-2	(66,68,73)
PLX4720 (BRAF^V600E^)	Rapamycin (mTOR) and PX-866 (PI3K)	↑ apoptosis	↓ pAkt, pS6, pMEK and pERK	(77)
Vemurafenib (BRAF^V600E^)	MK-2206 (Akt)	↓ cell proliferation, ↓ cell cycle progression, ↑ apoptosis	↓ pERK, pAkt, pS6K1 and cyclin D1, ↑ p27, Bim-E_L_ and cleaved-PARP	(51,80)
PLX4720 (BRAF^V600E^)	LY294002 (PI3K)	↑ apoptosis		(78)

4EBP1, eukaryotic translation initiation factor 4E binding protein 1; Akt, v-Akt murine thymoma viral oncogene; Bcl-2, B-cell lymphoma-2; cIAP-2, cellular inhibitor of apoptosis protein-2; ERK, extracellular signal-regulated kinase; bFGF, basic fibroblast growth factor; GSK3α/β, glycogen synthase kinase-3 α/β; HIF-1α, hypoxia-inducible factor-1-α; MEK, mitogen-activated protein/extracellular signal-regulated kinase; MMP2, matrix metallopeptidase 2; mTOR, mammalian target of rapamycin; PARP, poly(ADP-ribose) polymerase; PI3K, phosphatidylinositol-4,5-bisphosphate 3-kinase; S6K, ribosomal protein S6 kinase; VEGF, vascular endothelial growth factor.

**Table IV:** Pre-clinical studies combining RTKs targeting and other pro-apoptotic strategies.

Compound 1 (target 1)	Compound 2 (target 2)	Effect of combination vs. single treatments	Mechanism of synergy	Refs.
Sunitinib (RTKs)	Bortezomib (proteasome)	↓ cell viability, ↑ sub-G1 content	↑ cleaved-caspase-3	(100)
Dasatinib (RTKs)	U0126 (MEK1/2)	↑ cell death, ↓ invasion		(103)
Imatinib (RTKs)	TRAIL	↓ cell growth, ↑ apoptosis	↑ cleaved-caspase-8, -9, -3, Bax, ↓ c-FLIP, Bcl-2 and Bcl-X_L_, ↑ cytosolic Bcl-2 and cytochrome c, ↓ mitochondrial Bcl-2, Bcl-X_L_ and cytochrome c	(104)
Imatinib (RTKs)	Vatalanib (VEGFRs)	↑ response to paclitaxel *in vitro* (↓ cell proliferation) and *in vivo* (↓ tumor growth)		(105)
Bevacizumab (VEGF)	Rapamycin (mTOR)	↓ cell growth, cell loss		(106)
Vatalanib (VEGFRs)	Everolimus (mTOR)	↓ tumor growth and LN metastases	↓ plasma VEGF	(107)
Bevacizumab (VEGF)	Erlotinib (EGFR)	↓ tumor growth, LN and lung metastases	↓ proliferation and angiogenesis, ↑ apoptosis	(108)
Crizotinib (MET)	PD184352 (MEK) or PLX4720 (BRAF^V600E^)	↓ cell proliferation		(29)
SU11274 (MET)	Vemurafenib (BRAF^V600E^)	↓ cell growth and wound healing, ↑ G0/G1		(109)
PD166866 (FGFR) or SU5402 (FGFR) or dnFGFR	Sorafenib (multikinase/RAF)	↓ cell growth and ↑ apoptosis, ↓ tumor growth	↓ pERK, pAkt, pStat3	(90)
PD166866 (FGFR)	Vemurafenib (BRAF^V600E^)	↓ cell growth		(90)
Gefitinib (EGFR)	PLX4720 (BRAF^V600E^)	↓ cell growth and tumor growth		(110)
Lapatinib (ErbB2/EGFR)	PLX4720 (BRAF^V600E^)	↓ cell growth and tumor growth	↓ pAkt	(111)
Anti-ErbB3 mAb	Vemurafenib (BRAF^V600E^) or trametinib (MEK)	↓ cell growth	↓ pAkt, pERK	(112)
PPP (IGF-1R)	TRAIL	↑ sub-G1 content, ↓ cell survival		(113)
PPP (IGF-1R)	U0126 or trametinib (MEK)	↑ apoptosis and sub-G1 content	↓ pERK, pAkt, pBAD, Mcl-1	(114)

Akt, v-akt murine thymoma viral oncogene homolog; Bcl, B-cell lymphoma; cFLIP, cellular FLICE-inhibitory protein; EGFR, epidermal growth factor receptor; ERK, extracellular signal-regulated kinase; ErbB, v-erb-b2 avian erythroblastic leukemia viral oncogene homolog; FGFR, fibroblast growth factor receptor; IGF-1R, insulin-like growth factor-1 receptor; LN, lymph node; MEK, mitogen-activated protein/extracellular signal-regulated kinase; mTOR, mammalian target of rapamycin; PPP, cyclolignan picropodophyllin; RTKs, receptor tyrosine kinases; TRAIL, TNF-related apoptosis-inducing ligand; Stat, Signal transducers and activators of transcription; VEGF, vascular-endothelial growth factor; VEGFR, VEGF receptor.

**Table V:** Pre-clinical studies associating TRAIL and signaling pathway inhibition.

Compound 1 (target 1)	Compound 2 (target 2)	Effect of combination vs. single treatments	Mechanism of synergy	Refs.
U0126 (MEK1/2)	rhTRAIL (leucin zipper)	↑ apoptosis	↑ mitochondrial depolarization, ↑ Smac/DIABLO release, ↑ cleavage of caspase-3 and PARP	(122)
L-779,450 (pan-RAF), U0126 (MEK1/2) or vemurafenib (BRAF^V600E^)	Soluble TRAIL	↑ caspase-dependent apoptosis, ↓ cell proliferation	↑ mitochondrial depolarization, ↑ release of Smac/DIABLO, AIF and cytocrome c, ↑Bax, Bim-E_L_, ↑ caspase-9, -3 activity	(123)
Wortmannin (PI3K) or MK-2206 (Akt)	Soluble TRAIL	↑ apoptosis, ↓ cell proliferation	↑ ROS production, ↑ Bax activation	(124)
PP2, AZD0530 (Src kinase), PI103 (PI3K) or U0126 (MEK1/2)	rhTRAIL	↓ cell viability	↑ caspase-3, ↓ cell adhesion and motility	(125)
SBHA (HDAC)	Soluble TRAIL	↑ apoptosis	↑ mitochondrial apoptotic events, ↑ activation of Bax, Bak, Bid, Bim, ↓ Mcl-1, XIAP, Bcl-X_L_	(127,128)
Vorinostat (HDAC)	Ad-hTRAIL	↑ growth inhibition, ↑ cell death	↑ caspase-8, -9, -3 activation, ↑ Bid cleavage, ↑ loss of mitochondrial integrity, ↓ XIAP, Survivin, Bcl-X_L_, Mcl-1, ↑ TRAIL-Rs expression	(129)
AM404 (NFATc2)	Soluble TRAIL	↑ apoptosis		(130)
CHIR99021 (GSK-3)	rhTRAIL	↑ apoptosis	↓ AXIN-1	(131)
BMS-345541 (IKKβ)	Soluble TRAIL	↑ apoptosis	↑ Bax activation, ↓ XIAP, ↑ Smac/DIABLO	(132)
Bortezomib (proteasome), Smac mimetic-compound 3 (XIAP)	(iz)-TRAIL	↓ cell viability, ↑ apoptosis	↑ caspase-3, -8 activity, ↑ PARP cleavage	(133)
NPI-0052 or HMEQ (proteasome)	Soluble TRAIL	↑ apoptosis		(134)

Ad, adenoviral; AIF, apoptosis inducing factor; Akt, v-Akt murine thymoma viral oncogene; Bcl, B-cell lymphoma; cFLIP, cellular FLICE-inhibitory protein; cIAP, cellular inhibitor of apoptosis protein; CMV, cytomegalovirus; h, human; GSK, glycogen synthase kinase; HDAC, histone deacetylase; IKKβ, IkB kinase; (iz)-TRAIL, isoleucine zipper; mAb, monoclonal antibody; MEK, mitogen-activated protein/extracellular signal-regulated kinase; mTOR, mammalian target of rapamycin; rh, recombinant human; NFATc2, calcineurin/nuclear factor of activated T cells c2; PARP, poly(ADP-ribose) polymerase; PI3K, phosphatidylinositol-4,5-bisphosphate 3-kinase; ROS, reactive oxygen species; SBHA, suberoyl bis-hydroxamic acid; Smac/DIABLO, second mitochondria-derived activator of caspases/direct IAP-binding protein with low pI; TRAIL, TNF-related apoptosis-inducing ligand; TRAIL-R, TRAIL receptor; XIAP, X-linked inhibitor of apoptosis.

**Table VI:** Pre-clinical studies co-targeting anti-apoptotic proteins and the MAPK pathway.

Compound 1 (target 1)	Compound 2 (target 2)	Effect of combination vs. single treatments	Mechanism of synergy	Refs.
ABT-737 (anti-apoptotic Bcl-2 family molecules)	U0126 or PD0325901 (MEK)	↑ apoptosis, ↓ tumor growth	↑ Bim, ↓ Bcl-2 family members	(12)
ABT-737 (anti-apoptotic Bcl-2 family molecules)	PLX4720 (BRAF^V600E^)	↑ apoptosis, ↓ outgrowth of colonies	↑ PARP and caspase-9 and -3 cleavage, ↑ Bim, ↓ Mcl-1, ↑ mitochondrial membrane depolarization	(143)
ABT-737 (anti-apoptotic Bcl-2 family molecules)	PD98059 or CI-1040 (MEK)	↑ apoptosis		(144)
ABT-263 (anti-apoptotic Bcl-2 family molecules)	Selumetinib (MEK1/2)	↑ cell death		(146)
TW-37 (anti-apoptotic Bcl-2 family molecules)	U0126 or CI-1040 (MEK)	↑ apoptosis	Bim and p53 induction, ↓ Survivin, ↑ Bax/Bak, ↑ caspase-8, -9, -3 and -7 activation, ↑ cytochrome c and Smac/DIABLO release	(147)
Obatoclax (anti-apoptotic Bcl-2 family molecules)	PLX4720 (BRAF^V600E^)	↑ apoptosis, ↓ tumor volume	↓ Bcl2-A1	(149)
ABT-737 (anti-apoptotic Bcl-2 family molecules)	SB202190 or SB203580 (p38)	↑ apoptosis, ↓ cell viability	↑ caspase-8, -9 and -3 activation, ↑ Puma	(151)

Bcl, B-cell lymphoma; MEK, mitogen-activated protein/extracellular signal-regulated kinase; PARP, poly(ADP-ribose) polymerase; Smac/DIABLO, second mitochondria-derived activator of caspase/direct inhibitor of apoptosis-binding protein with low pI.

**Table VII:** Pre-clinical studies co-targeting proteasome, HDAC, anti-apoptotic molecules and/or survival pathways.

Compound 1 (target 1)	Compound 2 (target 2)	Effect of combination vs. single treatments	Mechanism of synergy	Refs.
Bortezomib (proteasome)	(−)gossypol (anti-apoptotic Bcl-2 family molecules)	↑ cell death, ↓ tumor growth and lung metastases	↑ cleaved-caspase-8, -9, -7, -3	(155)
Bortezomib (proteasome)	Obatoclax (anti-apoptotic Bcl-2 family molecules)	↑ apoptosis		(156)
Bortezomib (proteasome)	Rosiglitazone (PPAR-γ agonist)	↓ cell growth		(157)
Bortezomib (proteasome)	Smac mimetics (IAPs)	↓ cell viability	↑ cleaved PARP and cleaved caspase-3	(133)
MG-132 (proteasome)	ABT-737 (anti-apoptotic Bcl-2 familymolecules)	↓ cell viability, ↑ apoptosis	↑ Bak/Bax activation, ↑ cleaved caspase-3	(158)
Bortezomib (proteasome)	ABT-737 (anti-apoptotic Bcl-2 family molecules)	↓ cell viability, ↑ apoptosis, ↓ tumor growth		(159)
Bortezomib (proteasome)	LY294002 (PI3K)	↓ cell viability, ↑ sub-G0/G1	↑ cleaved caspase-3	(100)
BSc2118 (proteasome)	PD150606 (calpain)	↓ cell viability		(160)
Marizomib (proteasome)	Vorinostat (HDAC)	↓ cell growth, ↑ sub-G0/G1		(161)
Vorinostat (HDAC)	PLX4720 (BRAF^V600E^) or selumetinib (MEK1/2)	↑ apoptosis	↑ Bim-E_L_	(46)
Vorinostat (HDAC)	PLX4720 (BRAF^V600E^)	↓ cell viability, ↑ cell death (necrosis), ↓ tumor growth	↓ mitochondrial potential, ↑ Smac/DIABLO and cytochrome c release, ↑ cleaved PARP and caspase-9 and -3	(162)
CPA-7 (Stat3)	U0126 (MEK1/2) or SB590885 (BRAF)	↓ invasion, ↑ cell death		(103)
ABT-737 (anti-apoptotic Bcl-2 family molecules)	Obatoclax (anti-apoptotic Bcl-2 family molecules)	↑ apoptosis	↑ Bim	(156)
ABT-737 (anti-apoptotic Bcl-2 family molecules)	Noxa overexpression	↑ cell death		(167)
ABT-737 (anti-apoptotic Bcl-2 family molecules)	Maritoclax (Mcl-1)	↓ cell viability and colony-forming ability, ↑ apoptosis	↑ Bax activity, ↑ cleaved caspase-3	(168)

Bcl, B-cell lymphoma; HDAC, histone deacetylase; IAP, inhibitor of apoptosis; MEK, mitogen-activated protein/extracellular signal-regulated kinase; PARP, poly(ADP-ribose) polymerase; PI3K, phosphatidylinositol-4,5-bisphosphate 3-kinase; PPAR, peroxisome proliferator activated receptor; Smac/DIABLO, second mitochondria-derived activator of caspase/direct inhibitor of apoptosis-binding protein with low pI; Stat, signal transducers and activators of transcription; tBid, truncated Bid.

**Table VIII:** Clinical studies using combinatorial targeted therapy.

Compound 1	Compound 2	Phase	Clinical effect of dual treatment	Toxicity	Refs.
Temsirolimus (15–75 mg i.v. weekly)	Sorafenib (200 or 400 mg orally, qd or bid)	I	No CR or PR; 10/25 patients (40%) achieved SD. Median PFS was 2.1 mo	6/25 patients (24%) had grade-3 or -4 DLTs; 17 (68%) patients required dose reduction during the course of the treatment	(169)
Temsirolimus (25 mg i.v. weekly)	Sorafenib (200 mg orally bid)	II	The median PFS was 2.1 mo and median OS was 7 mo. Three/63 patients (5%) achieved PR	2 treatment-related deaths and an additional 4 patients (6%) with treatment-related grade-4 adverse events	(170)
Dabrafenib (150 mg orally bid)	Trametinib (1 or 2 mg orally qd)	I–II	Phase II results: median 9.4 mo PFS in combination group vs. 5.8 mo with dabrafenib monotherapy; 41 vs. 9% of patients alive and progression-free at 1 year; 76 vs. 54% CR or PR; 10.5 vs. 5.6 mo the median duration of response. Both patients with the BRAF^V600E^ mutation and those with the BRAF^V600K^ mutation had significant improvement in PFS	The MTD combination was not reached; the recommended phase II dose was combination 150/2. Reduced incidence of cutaneous squamous-cell carcinoma in patients receiving combination compared with dabrafenib monotherapy. One DLT	(172)
Bevacizumab (15 mg/kg i.v. every 21 days)	Everolimus (10 mg orally qd)	II	7/57 patients (12%) achieved major responses, 33 patients (58%) had SD at 6 weeks. The median PFS and OS were 4 and 8.6 mo, respectively. Approximately 43% of patients were alive after 12 mo of follow-up	Generally well tolerated; grade-3 toxicities in 25/57 patients (44%), no grade-4 toxicity. One death possibly related to treatment	(174)
Bevacizumab (10 mg i.v. every 2 weeks)	Temsirolimus (25 mg i.v. weekly)	II	PR in 3/17 patients (18%), SD at 8 weeks in 9 patients (53%). Maximal response duration for PR was 35 mo	Mostly well tolerated, but 2 grade-4 lymphopenia and 1 reversible grade-2 leukoencephalopathy	(175)
Tivantinib (360 mg orally bid)	Sorafenib (400 mg orally bid)	I	CR in 1/16 patients (6%), PR in 3 patients (19%), and 3 SD (19%). Overall response rate and disease control were 25 and 44%, respectively; median PFS was 5.3 mo	Well tolerated	Abs.^a^
Sorafenib (400 mg qd or bid, or 400 mg qam plus 200 mg qpm, orally)	Tipifarnib (100–300 mg orally qd, 3 weeks of every 4)	I	Three of the 7 melanoma patients (43%) had SD (4, 4 and 14 mo)	The most common DLT was grade-3 rash; the MTD was defined as sorafenib 400 mg qam plus 200 mg qpm; tipifarnib 100 mg bid	(171)
Sorafenib (400 qam plus 200 mg qpm, orally)	Tipifarnib (100 mg orally bid, 3 weeks of 4)	II	Median PFS 1.8 mo and OS 7 mo, with 1/39 patient (3%) achieving PR	1 treatment-related grade-4 toxicity	(170)
Sorafenib (400 mg orally bid, starting at day 14 before tanespimycin)	Tanespimycin (300–450 mg/m^2^ i.v. on days 1, 8 and 15 in a 28-day cycle)	I	Four of 6 (67%) patients with melanoma showed SD, mean duration 3.4 mo	DLT of grade-3 and -4 in one patient each were observed at 450 mg/m^2^ of tanespimycin. Recommended phase II doses are 400 mg sorafenib bid and 400 mg/m^2^ tanespimycin	(176)
Marizomib (0.15–0.7 mg/m^2^ i.v. on days 1, 8 and 15 of 28 day cycles)	Vorinostat (300 mg orally qd on days 1–16 of each cycle)	I	SD in 11/14 melanoma patients (79%); 4 of these patients maintained SD for ≥4 mo	No demonstration of unacceptable toxicity, with safety findings consistent with either drug alone	(161)

bid, twice daily; CR, complete response; DLTs, dose-limiting toxicities; mo, months; i.v., intravenously; MTD, maximum tolerated dose; PFS, progression free survival; PR, partial response; qam, every morning; qd, once a day; qpm, every evening; SD, stable disease; OS, overall survival.

a Means-Powell JA, *et al*, J Clin Oncol 30: 15, abs. 8519, 2012.
